# Modelling the effect of bednet coverage on malaria transmission in South Sudan

**DOI:** 10.1371/journal.pone.0198280

**Published:** 2018-06-07

**Authors:** Abdulaziz Y. A. Mukhtar, Justin B. Munyakazi, Rachid Ouifki, Allan E. Clark

**Affiliations:** 1 Department of Mathematics and Applied Mathematics, University of the Western Cape, Private Bag X17, Bellville 7535, South Africa; 2 DST-NRF Centre of Excellence in Mathematical and Statistical Sciences (CoE-Mass), University of the Witwatersrand, Private Bag 3, Wits 2050 Gauteng, South Africa; 3 Department of Mathematics and Applied Mathematics, Faculty of Natural & Agricultural Sciences, University of Pretoria, Private Bag X20, Hatfield 0028, South Africa; 4 Department of Statistical Sciences, University of Cape Town, Private Bag X3, Rondebosch 7701, Cape Town, South Africa; 5 Centre for Statistics in Ecology, Environment and Conservation (SEEC), University of Cape Town, Cape Town, South Africa; Institut Pasteur, FRANCE

## Abstract

A campaign for malaria control, using Long Lasting Insecticide Nets (LLINs) was launched in South Sudan in 2009. The success of such a campaign often depends upon adequate available resources and reliable surveillance data which help officials understand existing infections. An optimal allocation of resources for malaria control at a sub-national scale is therefore paramount to the success of efforts to reduce malaria prevalence. In this paper, we extend an existing SIR mathematical model to capture the effect of LLINs on malaria transmission. Available data on malaria is utilized to determine realistic parameter values of this model using a Bayesian approach via Markov Chain Monte Carlo (MCMC) methods. Then, we explore the parasite prevalence on a continued rollout of LLINs in three different settings in order to create a sub-national projection of malaria. Further, we calculate the model’s basic reproductive number and study its sensitivity to LLINs’ coverage and its efficacy. From the numerical simulation results, we notice a basic reproduction number, $R_{0}$, confirming a substantial increase of incidence cases if no form of intervention takes place in the community. This work indicates that an effective use of LLINs may reduce $R_{0}$ and hence malaria transmission. We hope that this study will provide a basis for recommending a scaling-up of the entry point of LLINs’ distribution that targets households in areas at risk of malaria.

## Introduction

The Republic of South Sudan (RSS) is among the countries in sub-Saharan Africa that are most severely affected by malaria and is currently experiencing an unprecedented outbreak of malaria. Médecins Sans Frontières (MSF) have reported that, in the year 2015, malaria outbreaks in South Sudan were considered to be the most hazardous in the region [1, 2]. The country is facing a number of tremendous challenges, the most notable being the limitation of human and financial resources due to the ongoing war and civilian instability. Nonetheless, the government agencies of South Sudan, as well as many Non-Governmental Organizations (NGOs) have committed to reducing this ongoing outbreak of malaria.

Recently, the National Malaria Control Program (NMCP) reported, in its strategic plan, that LLINs have been the main health intervention deployed to reduce malaria transmission in South Sudan since it gained independence [3]. A number of LLINs nets have been distributed since 2008, when the free mass LLIN distribution campaign was piloted in the States of Warrap, Western Bahr-El-Ghazal and Western Equatoria. However, their distribution and utilization still remain relatively low [4]. Subsequently, the programme was extended to the entire country reaching a total of 2 602 021 LLINs in 2009 [5, 6]. This number declined to 1 836 401 LLINs in 2011 and then, further down to 1 592 507 LLINs in 2012 in various states [7]. Moreover, in these campaigns ownership of LLINs by community members varied by State. For instance, Eastern Equatoria has the highest (58%) LLINs coverage, while the lowest coverage rates are found in Warap (17%), Unity (20%) and Upper Nile (22%). Likewise, malaria infection takes a larger toll in the rural areas where the availability of LLINs is slightly lower (31%) than in urban areas (44%) [8].

**Fig 1:**
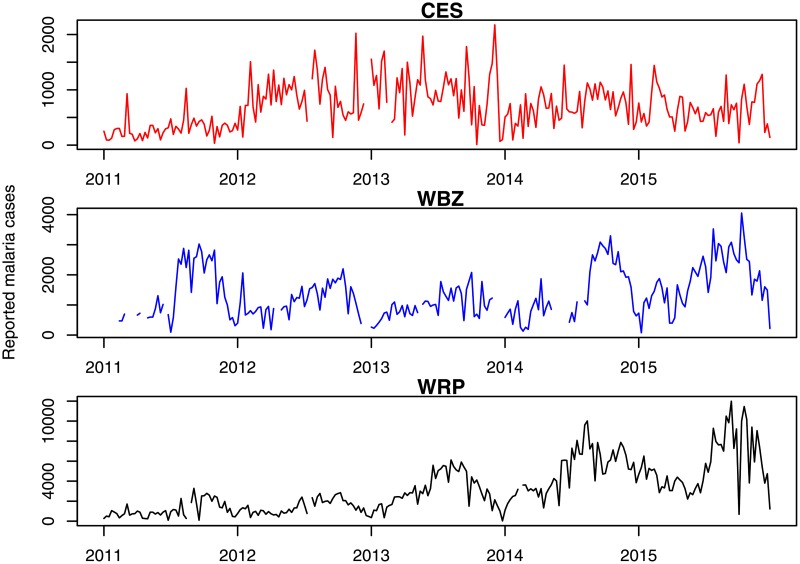
Reported weekly malaria cases between 2011 and 2015. Weekly malaria reported cases from the beginning of 2011 until end of 2015 in Central Equatoria State (CES), Western Bahr El Ghazal State (WBZ) and Warrap State (WRP). The data was obtained from National Malaria Control Programme of South Sudan (NMCP).

These control measures were not sufficient to eliminate the parasite over a short time scale and failed to sustain control programs. The malaria trend increased between 2011 and 2015 in almost all of the states, as is shown in Fig 1. This reported case data is accumulated on a weekly-basis. The data exhibits noise and some missing data is observed. Nonetheless, understanding the role of insecticide-treated nets in mosquito vectors is the first and most important step in disease eradication. Here, we focus on current malaria control actions and their impact on human infection. This will help to define the specific needs for successful malaria interventions in various settings, while increasing the impact of control tools and maintaining value for money. The key to effective control is to choose policies that are appropriate to local settings. The Ministry of Health (MoH) has endeavored to scale up malaria control efforts in the country in order to lower both the morbidity and mortality rates of malaria by 80% by the year 2020. As this 80% reduction in malaria prevalence may not be achieved through a ‘more of the same’ approach, mathematical modelling may play a role in operational strategies on control.

Mathematical models usually depend on a set of parameters. In the present case, each parameter carries a biological significance such as force of infection, recovery rate or, mortality rate. It is therefore important to evaluate the numerical values of these quantities with real data in order the computational simulation to predict the reactions accurately, and thus to give us a better understand of the disease epidemiology. In recent years there has been an increased interest in parameters determination procedures [9–11]. Bayesian approaches and in particular, the Markov Chain Monte Carlo (MCMC) have proven to be powerful inference tools for complex systems developed in behavioral science and computational biology [12–17].

Many mathematicians and epidemiologists (see for example, [18–26]) have provided different mathematical models for understanding the transmission dynamics of malaria in human populations and also for consolidating various intervention tools, such as LLINs, Indoor Residual Spraying (IRS), drugs and even vaccines. For example, Ngonghala et al. [25] developed a mathematical model for malaria dynamics that incorporates Insecticide-Treated Nets (ITNs) coverage. They concluded that when the reproduction number *R*_0_ < 1, the mosquito-free equilibrium is a globally asymptotically stable (GAS), whereas, when *R*_0_ is greater than one, a locally asymptotically stable human-mosquito equilibrium exists. Their study shows that constant ITNs-efficacy may underestimate the disease transmission risk. Chitnis et al. [26] adapted a mathematical model to compare the impact of malaria vector-control Interventions consist of ITNs and IRS, implemented individually and in combination; their results showed that ITNs were more effective than IRS. Okumu et al [27] used a deterministic model of the mosquito life cycle to investigate the effect of untreated nets or LLINs with IRS combinations on the disease at the community level, they concluded that the insecticidal potential impact of LLINs and IRS is mainly due to the personal protection provided by the nets, rather than insecticidal effectiveness. Briët and Penny [28] used a stochastic simulation model based on individuals in scenarios with sustained LLIN distributions, and varying degrees of Case Management (CM) coverage. The modelling analysis indicated that under sustained vector control and scaled-up CM, transmission can rebound to higher levels than when using LLIN distribution alone. Griffin et al. [29] developed an individual-based simulation model for Plasmodium falciparum transmission in an African context incorporating the impact of the switch to Artemisinin-Combination Therapy (ACT) and scaling up the coverage of interventions from the year 2000 onwards.

In this paper, we propose a modification of the age structured model developed by Filipe et al. [30], in the expression of the SEIR and SEI Model formulation for host and vector respectively. Our model does not include an age structure, but accounts for LLINs’ waning effect in order to forecast epidemiological aspects of malaria in South Sudan’s different regions and states. Consequently, a parameter estimation of this model is carried out under a Bayesian framework via Markov Chain Monte Carlo (MCMC) methods, wherein the likelihood function is combined with the prior values of the parameters in order to calculate the posterior values for model parameters from time series data.

## Study area and demography

South Sudan is a tropical landlocked country in East-Central Africa which shares borders with some of the most malaria-endemic countries in the world. Prior to 2015, the country was divided into ten states comprised of three regions.

**Fig 2:**
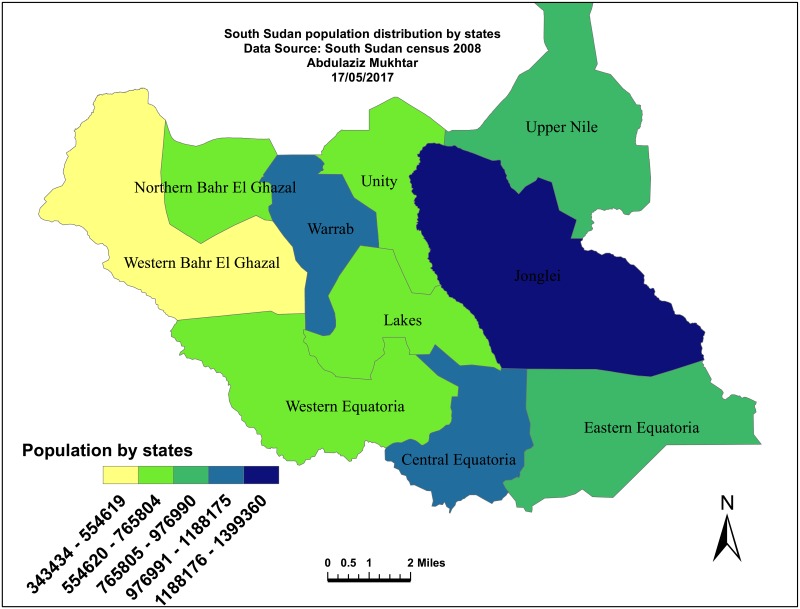
South Sudan population map. Population density of South Sudan 2009.

The pre-independence national census estimated the South Sudan population at 8.2 million people in 2008, with 42% of the population aged under 15 years, 19% at the median age and only 5% aged over 60 years [31]. The population projection for 2009 may be as high as 11 million due to both growth rates and the estimated numbers of returnees, Fig 2. The birth rate is estimated at 40.62 per thousand people and the maternal mortality rate is estimated at 1,700 deaths per 100,000 live births [32]. The country’s fertility rate of 6.7 births per woman is the highest in the Eastern Mediterranean region [32]. The census reported a life expectancy at birth of 42 years for both sexes [33]. This study is conducted in states chosen randomly in three different regions, namely:

1. Equatoria (South), we have chosen the state of Central Equatoria,
2. Bhar El Ghazal (North-west), we have chosen the state of Western Bahr El Ghazal,
3. Upper Nile (North-east), we have chosen the state of Warrap.

**Fig 3:**
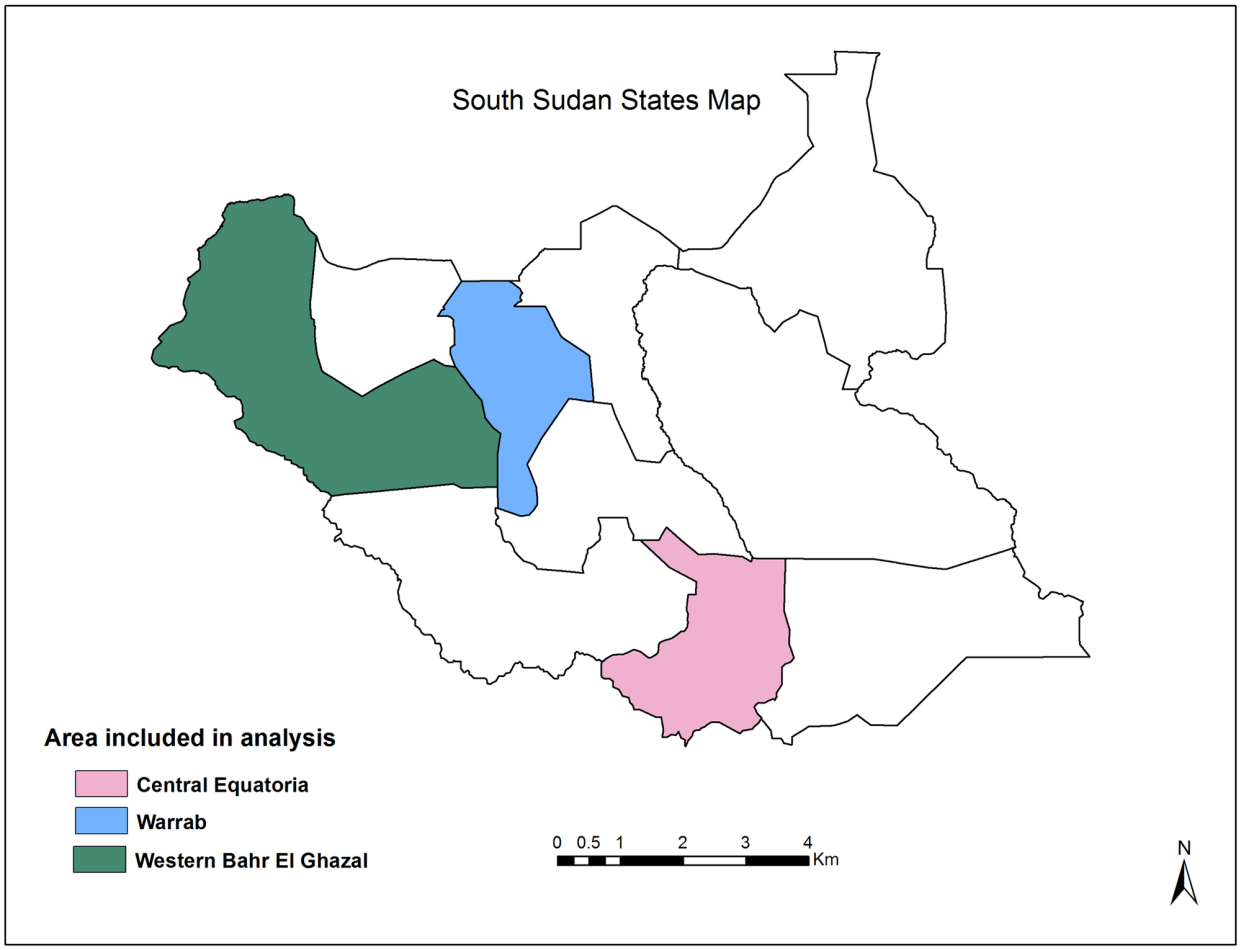
South Sudan selected state analysis map.

The map of the study area is given in Fig 3.

## Method

**Fig 4:**
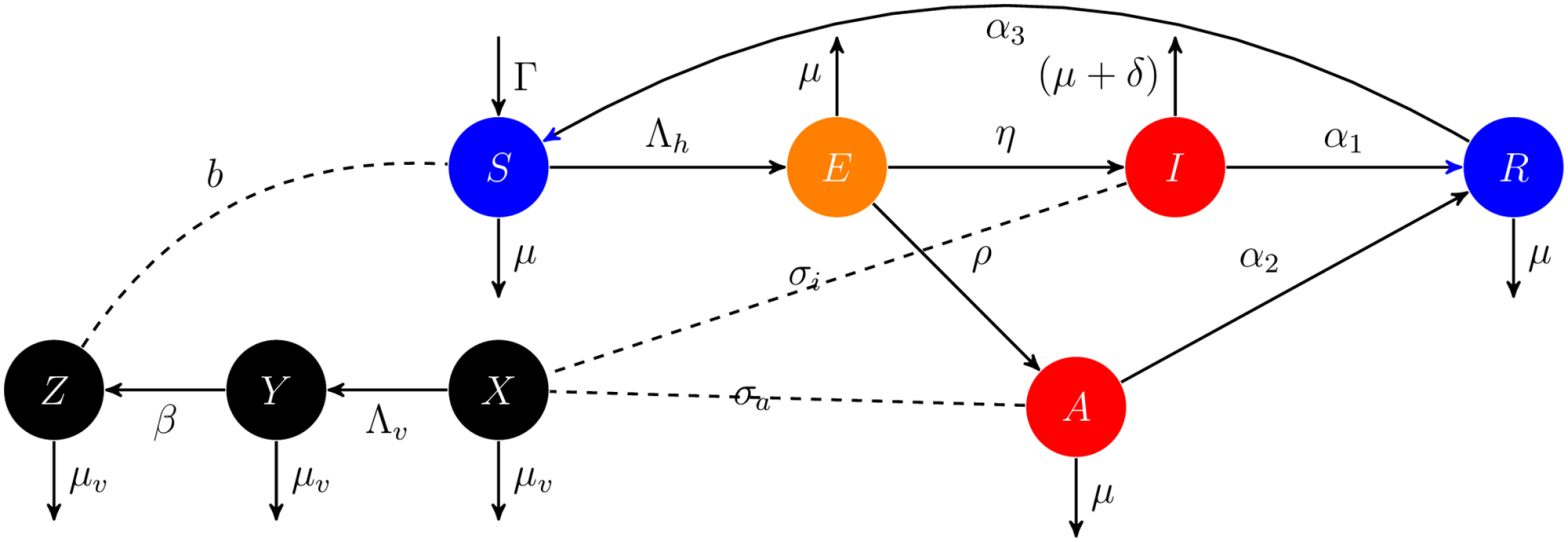
Malaria transmission model diagram. Flow diagram for Human and Mosquito infection model.

We use a deterministic compartmental structure for the endemic malaria disease. The total human population denoted by *N* is subdivided into susceptible individuals *S*, pre-infectious with malaria parasite individuals *E*, clinical infectious individuals *I*, asymptomatic infectious individuals *A* and protected individuals *R*, Eq (1). The total mosquito population denoted by *M* is subdivided into susceptible mosquitoes *X*, mosquitoes exposed to the malaria parasite *Y* and infectious mosquitoes *Z*, Eq (2). The compartmental model is illustrated in the flow digram in Fig 4, which translates to Eqs (3) and (6).

$$\begin{array}{ccc} N(t) & = & S(t)+E(t)+I(t)+A(t)+R(t), \end{array}$$ (1)

$$\begin{array}{ccc} M(t) & = & X(t)+Y(t)+Z(t). \end{array}$$ (2)

The human components of the model is presented to capture the relation of effective treatment and parasite prevalence. The second components of the model represent mosquito population dynamics to capture the effects of LLINs on vector mortality and in preventing transmission. The model excludes a delay in the force of infection and includes seasonality with respect to epidemics of this disease. We examine the model and data set, by applying Bayesian approach to estimate the posterior distribution of parameters. The prior estimates of some parameters were obtained from the literature [34, 35].

### Human model formulation

Since we are modelling the endemic malaria on a longer scale, we include birth and death rate in the model. Hence, the susceptible compartment is recruited by birth at a rate Γ. We presume susceptible individuals (*S*) acquire malaria and become infected at a rate Λ_*h*_ when they are bitten by infectious mosquitoes (the entomological inoculation rate, EIR). After bites from infectious mosquitoes, individuals progress to the pre-infectious compartment *E* and remain on average 12 days (latent period) before becoming fully infective. Upon infection, they either develop clinical infectious with probability rate *η* to enter compartment *I* or proceed with asymptomatic infection with probability rate *ρ* to enter compartment *A*. Those that develop disease (with symptoms) are successfully treated and naturally recovered with the rate *α*_1_ and subsequently enter a compartment of recovery (or a protected compartment *R*) which are assumed to lose immunity and move to the susceptible compartment at a rate of *α*_3_. Individuals with asymptomatic infection are assumed to recover naturally with a constant per ca-pita recovery rate *α*_2_ and enter *R* compartment. All compartments are stratified by level at which people are bitten by mosquitoes and also drop individuals at a natural death rate of $\mu =\frac{1}{q\times 360}$ *day*^−1^ where *q* is the human life expectancy in years. The deterministic model for the human dynamics is as follows

$$\begin{array}{c} \begin{array}{cc} \frac{dS}{dt} & =\Gamma -\Lambda_{h}S+\alpha_{3}R-\mu S,\\ \frac{dE}{dt} & =\Lambda_{h}S-\eta E-\rho E-\mu E,\\ \frac{dI}{dt} & =\eta E-(\alpha_{1}+\delta +\mu )I,\\ \frac{dA}{dt} & =\rho E-(\alpha_{2}+\mu )A,\\ \frac{dR}{dt} & =\alpha_{1}I+\alpha_{2}A-\alpha_{3}R-\mu R, \end{array} \end{array}$$ (3)

where t represents time and *α*_1_ and *α*_2_ symbolize human infection durations while *α*_3_ is depend on the loss of immunity duration. The force of infection, Eq (4) is assumed to vary by degree of exposure to mosquitoes due to geographic variation and is governed by the function

$$\begin{array}{c} \Lambda_{h}=EIR\;b=\left(\frac{\epsilon Z}{N}\right)b \end{array}$$ (4)

where EIR denotes the entomological inoculation rate, *b* is the probability of infection if bitten by an infectious mosquito, with 0 < *b* ≤ 1. The parameter *ϵ* is the per capita biting rate of mosquitoes to be measured for adults at study settings level.

### Mosquitoes model formulation

We consider Anopheles Gambiae mosquitoes which is the main anopheles species that transmits Plasmodium Falciparum in South Sudan [36]. The mosquito population is divided into three classes: susceptible *X*, latently infected *Y*, infectious *Z*. Susceptible female mosquitoes are recruited at the birth rate *Ψ*. We assume reduction in this compartment at the death rate *μ*_*v*_ and at the force of infection (see Eq (5)). Thus, the adult susceptible mosquito acquires malaria at a rate Λ_*v*_ which depends on the infectiousness of the human population:

$$\begin{array}{c} \Lambda_{v}=\frac{\sigma_{a}\epsilon A+\sigma_{i}\epsilon I}{N} \end{array}$$ (5)

where *σ*_*a*_ is the onward infectivity from an asymptomatic infectious and *σ*_*i*_ is the onward infectivity from a clinical infection. The parasite (in the form of gametocytes) enters the mosquito with some probability when the mosquito bites an infectious human and the mosquito moves from the susceptible to the infectious class at a rate determined by the force of infection. Once mosquitoes are infected, they pass through a latent period. Mosquitoes then become infectious to humans and remain infectious for life (until they die). They leave the population through a per ca-pita density-dependent natural death rate. The population dynamics and infection process of anopheles Gambiae mosquitoes are given by the following set of ordinary differential equations.

$$\begin{array}{c} \begin{array}{cc} \frac{dX}{dt} & =\Psi -\Lambda_{v}X-\mu_{v}X,\\ \frac{dY}{dt} & =\Lambda_{v}X-\left(\beta +\mu_{v}\right)Y,\\ \frac{dZ}{dt} & =\beta Y-\mu_{v}Z, \end{array} \end{array}$$ (6)

where *β* is the probability that a mosquito survives the extrinsic incubation period (EIP), *μ*_*v*_ is the death rate and Λ_*v*_ is the force of infection acting on mosquitoes.

## Basic reproductive number $R_{0}$

To determine the stability of this model we first evaluate the critical points of the model (3) and (6) of ODEs. The trivial critical point with no infected individuals is the point $E_{0}=\left(S^{*},E^{*},I^{*},A^{*},R^{*},X^{*},Y^{*},Z^{*}\right)=\left(\frac{\Gamma }{\mu },0,0,0,0,\frac{\Psi }{\mu_{v}},0,0\right)$. The basic reproduction number, denoted by $R_{0}$, plays a vital role in understanding the propagation of the relevant epidemic. It is defined as the average number of secondary infections that occur when one infective individual is introduced into a completely susceptible host population [37].

For the purpose of our model, the basic reproduction number of the models can be established by using the next generation matrix as presented in [37]. In Proposition 4.1 we compute the basic reproduction number for the system.

**Proposition 4.1**. *The basic reproduction number of the model* (3) *and* (6) *is*

$$\begin{array}{c} R_{0}=\sqrt{\frac{\epsilon^{2}b\beta \sigma_{a}\rho }{\mu_{v}\left(\beta +\mu_{v}\right)\left(\eta +\rho +\mu \right)\left(\mu +\alpha_{1}\right)}+\frac{\epsilon^{2}b\beta \sigma_{i}\eta }{\mu_{v}\left(\beta +\mu_{v}\right)\left(\eta +\rho +\mu \right)\left(\mu +\delta +\alpha_{2}\right)}} \end{array}$$ (7)

Proof: See S1 Appendix.

## Model fitting

In this section, our quantitative mathematical model is fitted to data. There are a few statistical techniques that are usually used to undertake parameter estimation when building a statistical model. In particular, maximum likelihood estimation (MLE) and Bayesian estimation are the most novel statistical tools used. As with the usage of MLE, a mathematical model that confronts a data can be influenced by the exact relationship between the parameters or by the complexity of the model [38, 39]. The Bayesian method combines the likelihood of the data as well as the prior distribution of the parameters of the model to obtain the posterior distribution of the parameters of the model. This allows one to make inference based on the posterior mean/ median of the parameters.

**Fig 5:**
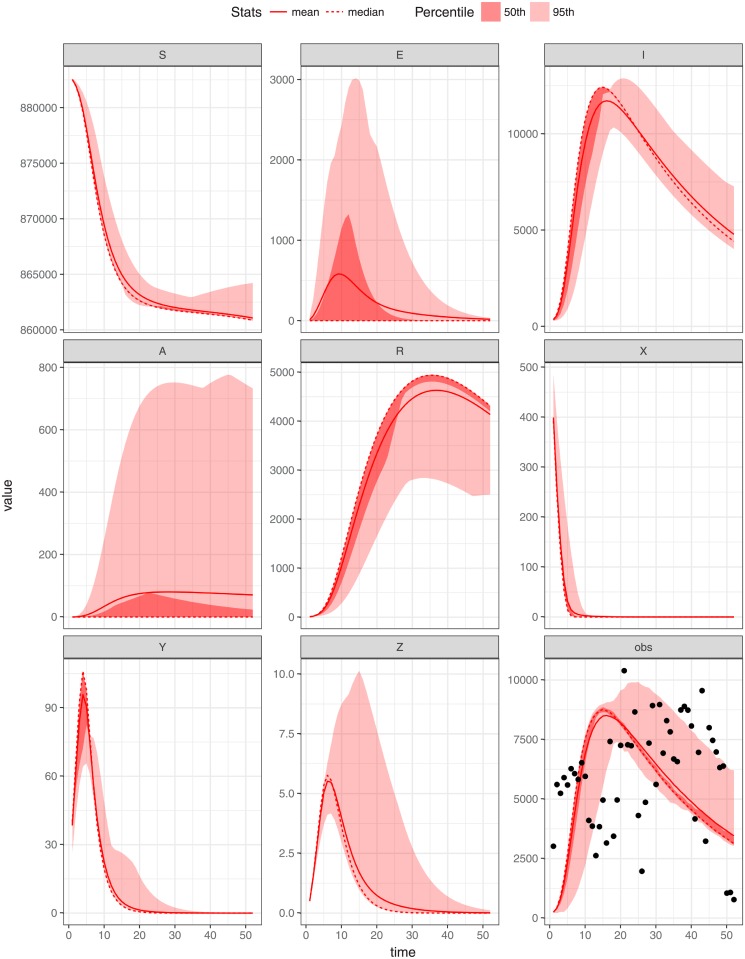
Parameters estimation by fitting model to weekly malaria cases of CES. The deterministic model (for both human and vector) trajectories and model assessment (lines) run against the CES data (points) with *ϵ* = 91.60, *b* = 0.4356804, *α*_1_ = 0.02169197 (duration of infections 46.1), *σ*_*i*_ = 0.6792, *α*_3_ = 0.5882 and initial state value (*S* = 882846, *E* = 0, *I* = 300, *A* = 0, *R* = 0, *X* = 600, *Y* = 0, *Z* = 0) with the mean and the median as well as the 95th and 50th percentiles of the replicated simulations are displayed.

**Fig 6:**
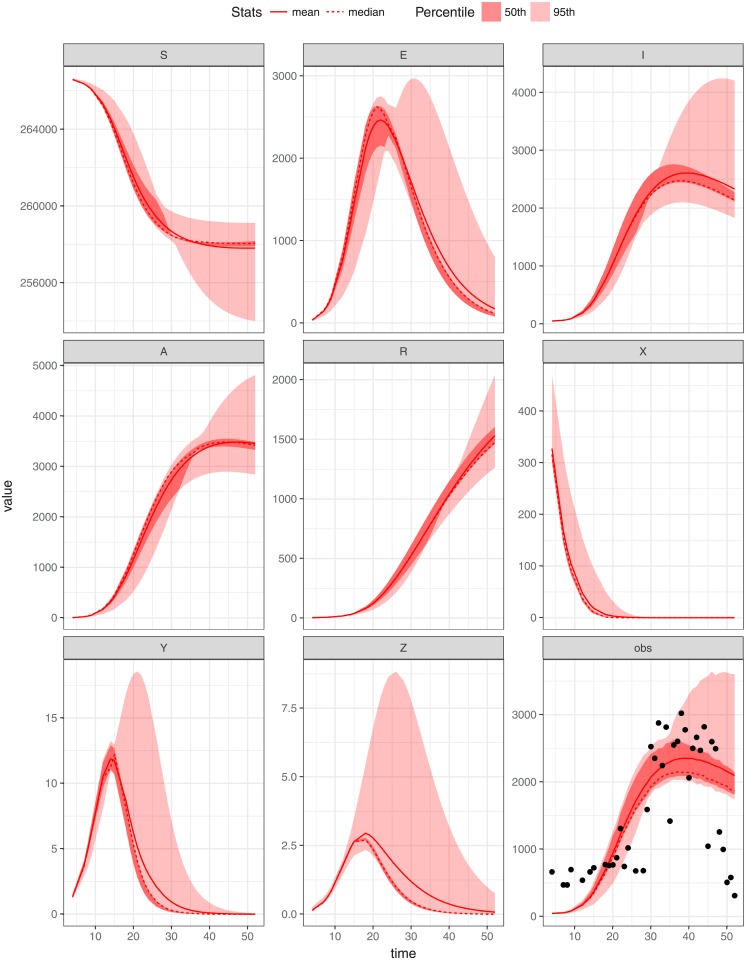
Parameters estimation by fitting model to weekly malaria cases of WBGZ. The deterministic model (for both human and vector) trajectories and model assessment (lines) run against the WBGZ data (points) for 2011, with *ϵ* = 79.50, *b* = 0.79836, *α*_1_ = 0.025, *α*_3_ = 0.01785714, *σ*_*i*_ = 0.06274 and initial state value (*S* = 266745, *E* = 0, *I* = 200, *A* = 0, *R* = 0, *X* = 500, *Y* = 0, *Z* = 0); the mean and the median as well as the 95th and 50th percentiles of the replicated simulations are displayed.

**Fig 7:**
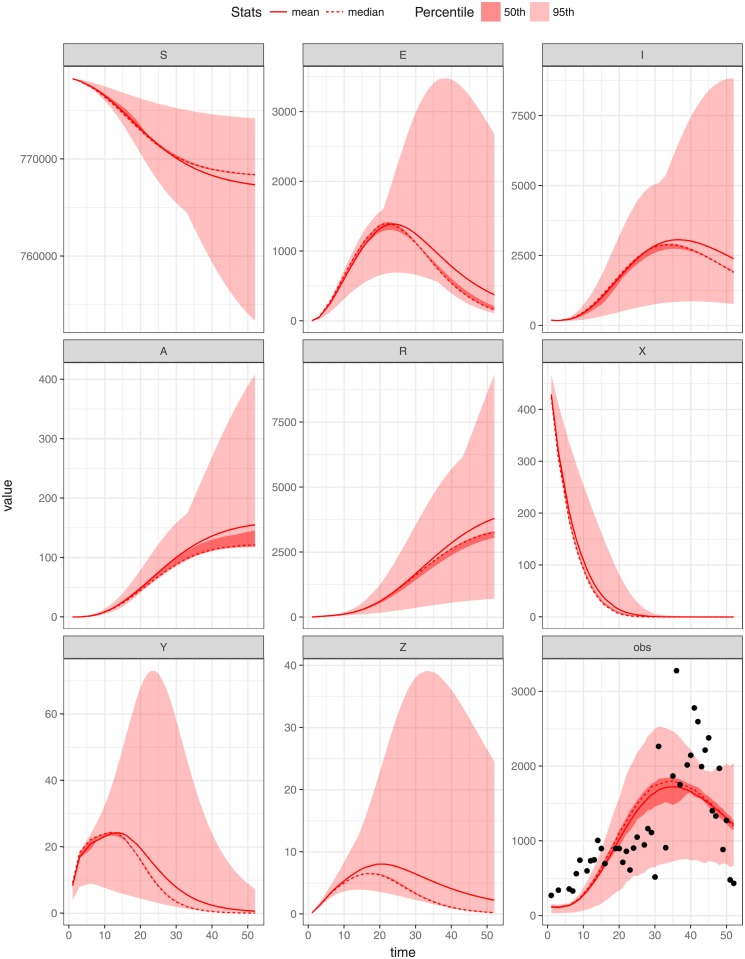
Parameters estimation by fitting model to weekly malaria cases of WRP. The deterministic model (for both human and vector) trajectories and model assessment (lines) run against the WRP data (points), with *ϵ* = 56.0, *b* = 0.63984, *α*_1_ = 0.0191938, *α*_3_ = 0.05, *σ*_*i*_ = 0.789852 and initial state value (*S* = 778342, *E* = 0, *I* = 200, *A* = 0, *R* = 0, *X* = 500, *Y* = 0, *Z* = 0); the mean and the median as well as the 95th and 50th percentiles of the replicated simulations are displayed.

In this study we utilise Markov Chain Monte Carlo (MCMC) to obtain the posterior samples of the parameters of the model. The model fitting was undertaken by using weekly malaria data of 2011 for each region under investigation (shown in Figs 5–7) using MCMC. The model is run from the year 2000 to reach a steady state before being fitted to data from the year 2011. We assume that weekly malaria data were reported according to a Poisson process with reporting rate *γ*. Since the reporting rate is unknown we assume it to be no larger than 85%. Assume also that *x*_*ij*_(*i* = 1, …, *n*; *j* = 1, …, 3) are the observed weekly malaria cases for state *j* during week *i*. We used uniform distributions to model the prior belief regarding the mosquito biting rate *ϵ* and the clinical duration of infections *α*_1_. Specifically we assume *ϵ* ∼ *U*(0, 200) and *α*_1_ ∼ *U*(0, 50) [34]. During this fitting process the model parameters described in Table 1, *b*, *ϵ*_*j*_, *α*_1_, and *α*_3_ were estimated and *δ*, *α*_2_, *ρ* and *η* were fixed (in agreement with previous studies) in each setting. These parameters were assumed to be constant and were jointly estimated by utilizing fitR (version 0.1 [40]) to obtain posterior samples 10000 iterations and a burn-in of 1000 iterations used for three chains. The credibility intervals produced in Figs 5–7 was a 95% confidence intervals with different accepting rate of each figure. It seems as if the model does not fit the weekly malaria data very well since the seasonality observed in the data has not been accounted for. Below we attempt to do so.

**Table 1:** Model parameters: Description and value.

Symbol	Description	Estimate	Ref
*μ*	Natural death rate of humans	0.00006614	Est
*σ*_*a*_	Onward infectivity from an asymptomatic infectious	0.2	[19]
*ρ*	Probability of asymptomatic infectious	0.0071 (fixed)	[19]
*α*_2_	Asymptomatic infection rate	1/200 (1/180–1/250) fixed	[42]
*δ*	Humans death rate due to malaria	0.0004 (0.00027-0.0005) fixed	[18]
*η*	Probability of acquiring clinical disease	1/12 (fixed) day^−1^	[42]
Γ	Birth rate of humans	Humans/Day	Est
*σ*_*i*_	Onward infectivity from a clinical infectious	Derived from data	
*b*	Probability of infection	Derived from data	
*ϵ*_*j*_	Mosquitoes biting rate for state *j*	Derived from data	
*α*_1_	Clinical disease rate	Derived from data	
*α*_3_	Human Re-susceptibility rate	Based on drug	

Parameters description and value driving the mathematical model of malaria.

**Fig 8:**
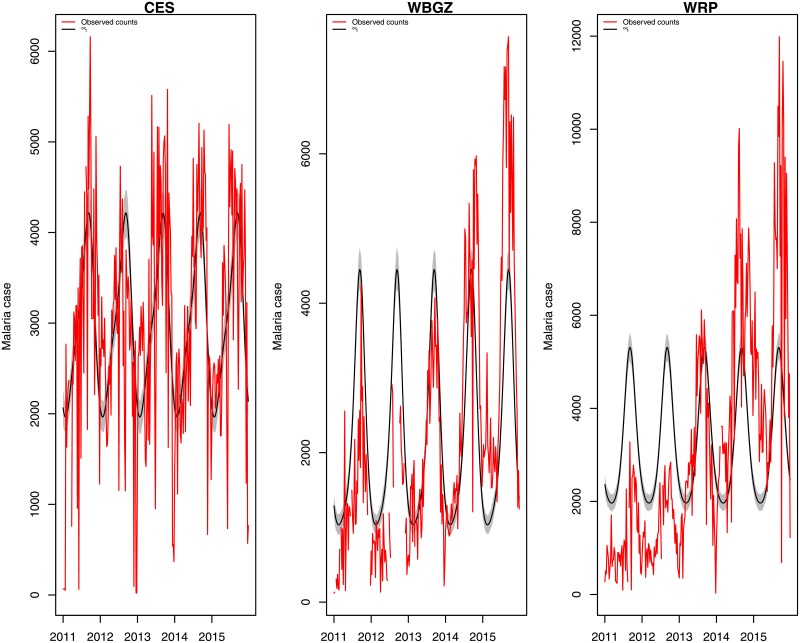
Model validation by fitting to malaria cases on weekely basis between 2011-2015. The posterior mean densities of the mosquito bite rate *ϵ* in CES, WBGZ and WRP with 95% credibility interval at a chosen number of 10,000 iterations.

In the second model, we calculate the mean of postorier distribution for further validation and better fitting results, using weekly malaria data between 2011 to 2015 (see S1 Dataset) plotted in Fig 8. This includes a simple parametric model to account for seasonality, as the weekly malaria count displayed strong seasonality. We specifically assume that the seasonal component is modelled as

$$\begin{array}{c} \beta_{0t}=\sum_{j=1}^{2}a_{j}\cos\left(tw_{j}\right)+b_{j}\sin\left(tw_{j}\right), \end{array}$$ (8)

where *w*_*j*_ = 2*πj*/52 and *t* represents time. We assume that the prior distribution of *a*_*j*_ and *b*_*j*_ are both Gaussian random variables with mean 0 and variance *σ*_*j*_ = 1.67001 [34]. For a given set of parameters, let the model-predicted malaria in site *j* be *θ*_*j*_, the number of initial susceptible individuals at risk and number of malaria cases be *E*_*j*_ and *x*_*ij*_ respectively. The hierarchical model used to validate the model for the observed malaria counts is thus:

$$\begin{array}{c} x_{ij}|\theta_{j},\beta_{0}∼\text{Poisson}\left(E_{j}e^{\beta_{0t}}\theta_{j}\right),\\ \begin{array}{c} \theta_{i}∼U\left(l_{1},l_{2}\right), \end{array} \end{array}$$

where *l*_1_ and *l*_2_ are known constants. The *E*_*j*_ values were obtained using [32].

## Intervention with bednets (LLINs)

The LLINs is the only intervention used in the study and hence, by testing its scale up effect on malaria transmission. We use data of LLINs coverage at state level for 2009 as baseline calculated from the given number of LLIN distribution which is provided by MIS and has been presented in [32]. In our model, LLINs intervention results (shown in Table 2) in:

1. Mosquito biting rate: A reduction of the mosquito biting rate is given by
  $$\begin{array}{c} (1-\chi V)\epsilon \end{array}$$
  where *χ* is the proportion of LLINs coverage and *V* is the effectiveness of LLINs. For the sake of calculating the basic reproduction number of the model with LLINs intervention, we assume that *V* is constant. However, in simulation practice, we consider the efficacy of LLINs wanes with time since mass distribution of LLINs campaigns were run in just one to four states in any single year. To account for this, we use in our model simulations, the following formula taken from [41].
  $$\begin{array}{c} V\left(t\right)=1-a\exp\left(-e^{-x(t-y)}\right) \end{array}$$ (9)
  where *a* and *x* are real numbers and *y* is a positive integer.
2. Mosquito mortality rate: LLINs intervention is also assumed to have three effects on the adult mosquito population. Firstly, increase the overall mosquito death that land on the nets. Secondly, repelling and possibly diverting mosquitoes to an animal blood host due to either insecticide irritation or the physical barrier of the net. Thirdly, lengthening the duration of the gonotrophic cycle leading to a reduced oviposition rate. Interested readers may also consult [42, 43] for further details.
  The probability that a surviving mosquito succeeds in feeding during a single attempt is given by
  $$\begin{array}{c} W=1+\chi \phi (s-1) \end{array}$$ (10)
  where *ϕ* is the proportion of people in bed when they are bitten and *s* is the probability of a mosquito feeding successfully on a person sleeping under a bed net.
  The probability of a mosquito being repelled without feeding is thus
  $$\begin{array}{c} Z(\chi )=\phi \chi r. \end{array}$$ (11)
  where *r* is probability of a mosquito being repelled by a bed net. At *χ* LLIN coverage, the duration of a feeding cycle is given by 1/*f*(*χ*) = *τ*_1_/(1 − *Z*)+ *τ*_2_ where *τ*_1_ is the time spent searching for a blood meal and *τ*_2_ is the time spent resting which is unaffected with intervention.
  Thus, the probability of a mosquito surviving one feeding cycle is given by
  $$\begin{array}{c} \beta \left(\chi \right)={\left(\frac{\beta_{1}\beta_{2}W}{1-Z\beta_{1}}\right)}^{f(\chi )} \end{array}$$ (12)
  where $\beta_{1}\;=\;e^{-\mu \left(0\right)\tau_{1}}$ and $\beta_{2}\;=\;e^{-\mu \left(0\right)\tau_{2}}$ are the probability of a mosquito surviving the periods of feeding and resting.
  The mosquitoes mortality which depend on bed nets coverage is thus
  $$\begin{array}{c} \mu_{v}\left(\chi \right)=-\log\beta \left(\chi \right) \end{array}$$ (13)

**Table 2:** Intervention parameters.

Symbol	Description	Estimate	Ref
Ψ	Per ca-pita birth rate of mosquitoes	0.13	[26]
*χ*	LLIN coverage	Est from data	
*f*	Inverse of gonotrophic cycle	1/3 day^−1^	[29]
*ϕ*	Proportion of bites taken on humans when in bed	0.89	[29]
*s*	Probability that a mosquito feeds successfully by a bed net	0.03	[34]
*r*	Probability of a mosquito being repelled by a bed net	0.56	[42]
*τ*_1_	Time spent seeking blood meal during gonotrophic cycle	0.69 days	[29]
*τ*_2_	Time spent resting during gonotrophic cycle	2.31 days	[34]
*β*_1_	Probability of a mosquito surviving the periods of feeding	0.91	[34]
*β*_2_	the probability of a mosquito surviving the periods of resting	0.74	[34]
*V*(*t*)	The efficacy of LLIN	Eq (9)	[41]
*W*(*χ*)	Probability of mosquito successfully feeding	Eq (10)	[42]
*Z*(*χ*)	Probability of mosquito repeating	Eq (11)	[42]
*β*(*χ*)	Daily survival probability	Eq (12)	[42]
*μ*_*v*_(*χ*)	Daily mosquito mortality	Eq (13)	[42]

Parameter values used in simulation and their sources.

On introducing the use of LLINs, $R_{0}$ becomes

$$\begin{array}{c} R_{0}\left(\chi \right)\;=\;\sqrt{\frac{{\left(1-V\chi \right)}^{2}\epsilon^{2}b\beta \left(\chi \right)\sigma_{a}\rho }{\mu_{v}\left(\chi \right)\left(\beta \left(\chi \right)\;+\;\mu_{v}\left(\chi \right)\right)\left(\eta \;+\;\rho \;+\;\mu \right)\left(\mu \;+\;\alpha_{1}\right)\;}+\;\frac{{\left(1-V\chi \right)}^{2}\epsilon^{2}b\beta \left(\chi \right)\sigma_{i}\eta }{\mu_{v}\left(\chi \right)\left(\beta \left(\chi \right)\;+\;\mu_{v}\left(\chi \right)\right)\left(\eta \;+\;\rho \;+\;\mu \right)\left(\mu \;+\;\delta \;+\;\alpha_{2}\right)}} \end{array}$$ (14)

We will now analyse the sensitivity index of $R_{0}$ with respect to the parameters *χ*, *V* and *ϵ* according to the definition below.

**Definition 1**. *The sensitivity index of* $R_{0}$ *with respect to a parameter p is given by*

$$\begin{array}{c} \Gamma_{R_{0}}^{p}=\frac{\partial R_{0}}{\partial p}\frac{p}{R_{0}}. \end{array}$$

Rewriting Eq (14) as

$$\begin{array}{c} R_{0}\left(\chi \right)=C\epsilon \left(1-V\chi \right)g\left(\chi \right), \end{array}$$ (15)

where

$$\begin{array}{ccc} C & = & \sqrt{\frac{b}{(\eta \;+\rho +\mu }[\frac{\sigma_{a}\rho }{\mu +\alpha_{2}}+\frac{\sigma_{i}\eta }{\mu +\delta +\alpha_{1}}]} \end{array}$$

and

$$\begin{array}{ccc} g(\chi ) & = & \sqrt{\frac{\beta (\chi )}{\mu_{v}\left(\chi \right)\left(\beta \left(\chi \right)+\mu_{v}\left(\chi \right)\right)}} \end{array}$$

and using the definition above, we have:

- The sensitivity index with respect to *ϵ* is
  $$\begin{array}{c} \Gamma_{R_{0}}^{\epsilon }=\frac{\partial R_{0}}{\partial \epsilon }\frac{\epsilon }{R_{0}}=\frac{C\epsilon (1-V\chi )g(\chi )}{R_{0}}=1. \end{array}$$
  This means that 10% increase (reductions) in *ϵ* would results in 10% increase (reductions) in $R_{0}$.
- The sensitivity index with respect to *V* is
  $$\begin{array}{c} \Gamma_{R_{0}}^{V}=\frac{\partial R_{0}}{\partial V}\frac{V}{R_{0}}=-C\chi g\left(\chi \right)\frac{V}{C\epsilon (1-V\chi )g(\chi )}=\frac{-V\chi }{C(1-V\chi )}. \end{array}$$
  This means that 10% increase (reductions) in *V* would result in $\frac{10V\chi }{C(1-V\chi )}\%$ increase (reductions) in $R_{0}$.
- The sensitivity index with respect to *χ* is
  $$\begin{array}{c} \Gamma_{R_{0}}^{\chi }=\frac{\partial R_{0}}{\partial \chi }\frac{\chi }{R_{0}}=\left(1-V\chi \right){\left(g\left(\chi \right)\right)}^{\prime }\frac{\chi }{C\epsilon (1-V\chi )g(\chi )}=\frac{\chi (-Vg\left(\chi \right)+\left(1-V\chi \right){\left(g\left(\chi \right)\right)}^{\prime })}{C\epsilon (1-V\chi )g(\chi )}. \end{array}$$
  This means that 10% increase (reductions) in *χ* would result in $\frac{10\chi (-Vg\left(\chi \right)+\left(1-V\chi \right){\left(g\left(\chi \right)\right)}^{\prime })}{C\epsilon (1-V\chi )g(\chi )}\%$ increase (reductions) in $R_{0}$.

It is to be noted that we have omitted the explicit expression of *g*′(*χ*) to avoid presenting long mathematical derivations.

## Results

**Fig 9:**
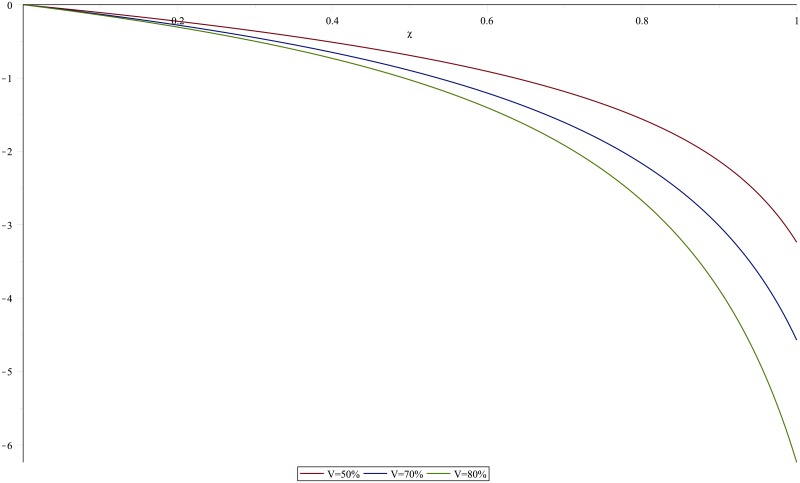
Sensitivity index of $R_{0}$ with respect to *χ*. Sensitivity index of $R_{0}$ with respect to *χ* for different values of *V*.

**Fig 10:**
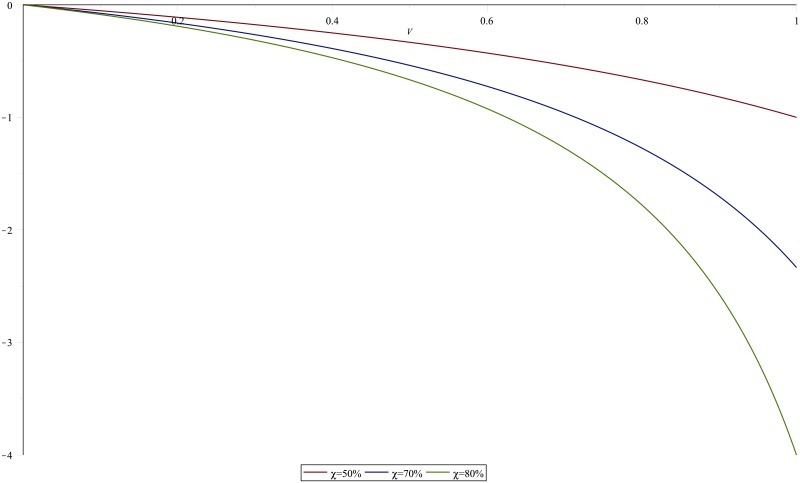
Sensitivity index of $R_{0}$ with respect to *V*. Sensitivity index of $R_{0}$ with respect to *V* for different values of *χ*.

The model fitting results are presented first before evaluating the predicted partial impact of the reduction-focused LLINs interventions. We simulate the model trajectory showing the population dynamics of humans expressed in susceptible, pre-infectious, clinical infectious, asymptomatic infectious, and in recovery compartments and vector population dynamics while simultaneously fitting the infectious class to data, using package fitR (version 0.1 [40]). The model does display some misfit due to missing values and the simplicity of the basic model. In Fig 8 model parameters estimated are *b*, *ϵ*_*j*_, *α*_1_, and *α*_3_, again projected to five years of data after a run in order to reach a steady state, incorporating a seasonal model. We carried out a sensitivity analysis of different parameters: all parameters are fixed and one is left to hold different values (+/- 10%) so that its influence on the system behaviour can be assessed. We found that the disease transmission increases or decreases greatly with an increase or decrease in the contact rate to susceptible mosquito *σ*_*i*_ and the biting rate *ϵ*. We also observed that, a longer infection period 1/*α*_1_ enhances disease transmission, which may lead to an increased contact rate to susceptible mosquitoes. To check the accuracy of our results, we ran the model using various sets of parameter values (independent chains) and tested whether individual distributions converge to the expected parameter value. Indeed, we found that the parameter sets converged to the posterior parameter values. Furthermore, on different occasions, the reproduction number, $R_{0}$ is the parameter most sensitive to the biting rate. For instance, $R_{0}$ reaches 15 when the bite rate is 117 per person per year but it is reduced to 6 when the bite rate is 34 per person per year, as obtained using Eq (7). This means that the prevalence of malaria will increase with an increase in the corresponding rate of mosquito bites and it will decrease with optimal mosquito control. The sensitivity index of $R_{0}$ with respect to *χ* and *V* are simulated in Figs 9 and 10 respectively where the parameter values from Tables 1 and 2 were used.

**Fig 11:**
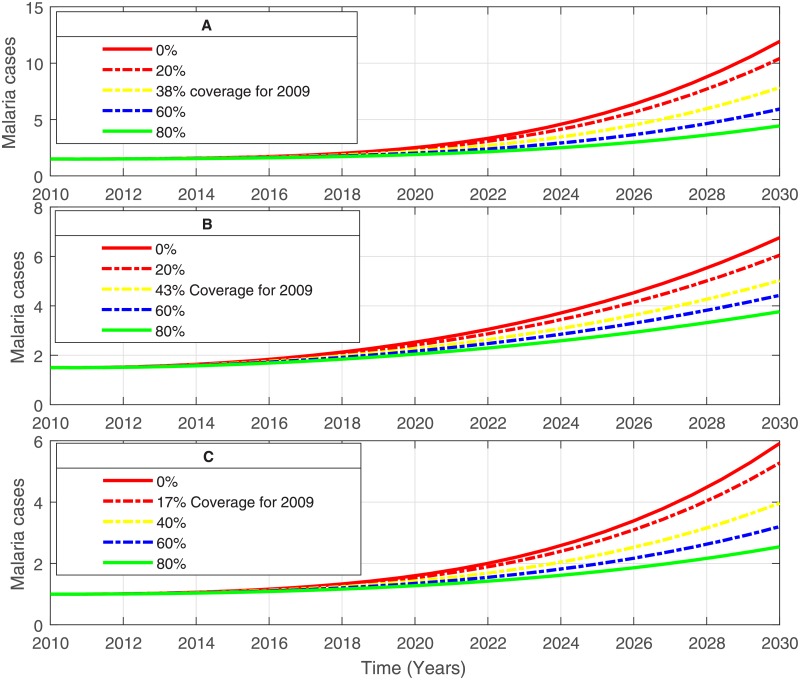
Prediction of malaria infections. Projected cases of malaria in hundred thousands of people with: No interventions of LLINs, coverage based on 2009 LLINs distribution, and additional coverage of LLINs; (A) in Central Equatoria, (B) in Western Bahr-El-Ghazal and (C) in Warrap.

The model’s key parameters are estimated through data-fitting procedures and along with those presented in Tables 1 and 2 are used to project the disease. The model results were relatively robust to variations in the long lasting efficacy of LLINs which decrease the biting rate and increase the mosquito mortality rate. The predicted potential impact of the use of LLINs by humans as an intervention strategy for combatting malaria in the three study areas (different states in different regions) is illustrated in Fig 11. For instance, a slight change in LLINs coverage can drastically affect the lifespan and hence the patterns of mosquito bites.

**Fig 12:**
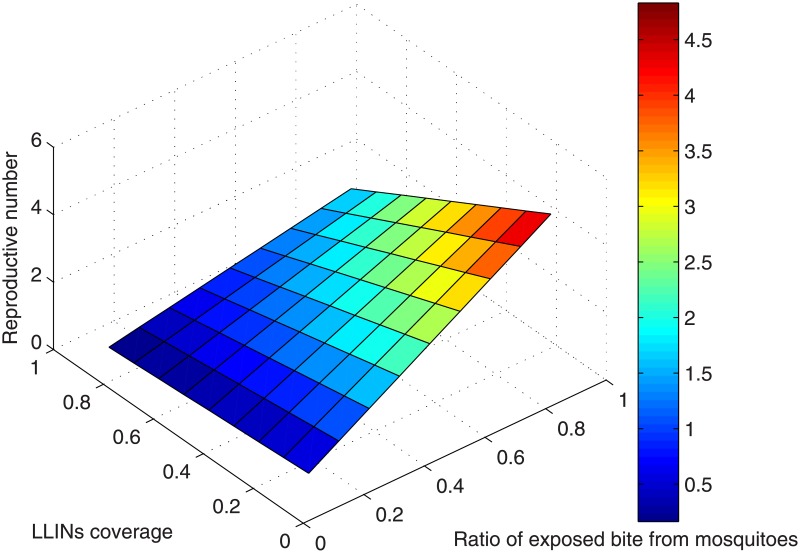
Prediction of basic reproduction number. Reproduction number as a function of Mosquito bite rate and LLINs coverage, we observe that the optimum coverage window for falciparum malaria transmission is 80–90.

We further derived and examined the basic reproduction number in relation to biteing rate values and LLINs coverage from Eq (14) plotted in Fig 12. With the low transmission of a bites rate of two infectious bites per person per year, the proportion of infections resulting in the reproduction number falls to less than one with the impact of intervention seemingly higher. But with more infectious bites per person per year LLINs coverage alone has less impact and may not succeed in reducing the reproduction number to less than one. Measuring the basic reproduction number can be difficult, but it can also be the most direct measurement for examining the effect of vector control interventions.

## Conclusion

In this paper, we presented a mathematical model in order to explore the impact of LLINs on malaria transmissions using a system of ordinary differential equations. The model analysis was based on a modification of a host-vector model presented by Filipe et al [30]. The Bayesian framework was incorporated to provide a posterior distribution of the parameters of the model given the malaria trial data. The threshold parameter, $R_{0}$, is the number of humans and mosquitoes expected to be infected with malaria by a single infected individual/mosquito introduced into a naive population. It was computed using the next ge neration matrix method. Owing to the reproduction number’s sensitivity to the mosquitoes biting rate, it is reasonable to recommend the use of LLINs as intervention strategy for malaria transmission. Therefore, we modelled the intervention to behave like a function comprised of three parameters: the proportion of LLIN coverage, the proportion of individuals exposed to mosquito bites, and the effectiveness of the nets. Simulation results of $R_{0}$ show that the use of bed nets with long term effectiveness could reduces $R_{0}$ to less than one in low transmission sites (at a bites rate of two infectious bites per person per year). In the absence of any intervention, we note a large number of $R_{0}$, confirming a substantial increase in incidence of malaria in the community. We cannot be sure whether the coverage of LLINs could eradicate malaria in an equivalent setting. Nevertheless, in low-transmission areas, LLINs have the ability to reduce malaria transmission to low levels, provided the interventions have high-use levels. Meanwhile, in moderate and high-transmission in these selected settings there was little change in the incidence levels. Thus, in these settings, novel tools and/or substantial social improvements might be required to achieve considerable reductions in malaria prevalence. Finally, the model is useful for further understanding future cases of malaria in South Sudan. This work shows that the use of LLINs with long term effectiveness may reduce $R_{0}$ and hence malaria transmission.

## Supporting information

S1 Appendix Proof of the proportion 4.1. (PDF)

S1 Dataset Weekly malaria cases data. (CSV)

## References

[1] Nguyen C, (2016) Malaria Ravages South Sudan. Available: http://www.healthmap.org/site/diseasedaily/article/malaria-ravages-south-sudan-11016. Accessed 10 January 2016.

[2] Cornelioa CO and Seriano OF. Malaria in South Sudan 1: introduction and pathophysiology. Southern Sudan Medical Journal. 2011; 4:7–9.

[3] Eyobo MB, Awur AC, Wani G, Julla A, Remijo CD, Sebit B, et al Malaria indicator survey 2009, South Sudan: baseline results at household level. Malaria Journal. 2014; 13: 13–45. doi: 10.1186/1475-2875-13-45 24490895 10.1186/1475-2875-13-45 PMC3922095

[4] Chanda E, Doggale C, Pasquale H, Azairwe R, Baba S, Mnzava A. Addressing malaria vector control challenges in South Sudan: proposed recommendations. Malaria Journal. 2013; 12:12–59. doi: 10.1186/1475-2875-12-59 23394124 10.1186/1475-2875-12-59 PMC3570318

[5] Pasquale H, Jarvese M, Julla A, Doggale C, Sebit B, Lual MY, et al Malaria control in South Sudan, 2006-2013: Strategies progress and challenges. Malaria Journal. 2013; 12: 1475–2875. doi: 10.1186/1475-2875-12-374 10.1186/1475-2875-12-374 PMC3816306 24160336

[6] Chanda E, Remijo CD, Pasquale H, Babab SP, Lako RL. Scale-up of a programme for malaria vector control using long-lasting insecticide-treated nets: lessons from South Sudan. Bulletin World Health Organization. 2014; 92: 290–296. doi: 10.2471/BLT.13.126862 10.2471/BLT.13.126862 PMC3967576 24700997

[7] GoSS: Malaria control strategic plan 2006–2011. Juba: Government of Southern Sudan. Ministry of Health; 2006.

[8] Manoja L. Health sector development plan 2011-2015. Government of South Sudan: Minister of health 2011 3 1.

[9] Huppert A, and Katriel G. Mathematical modelling and prediction in infectious disease epidemiology. Clin Microbiol Infect. 2013; 19: 999–1005. doi: 10.1111/1469-0691.12308 24266045 10.1111/1469-0691.12308

[10] Silal SP, Little F, Barnes KI, White LJ. Towards malaria elimination in Mpumalanga, South Africa: a population-level mathematical modelling approach. Malaria Journal. 2014; 13: 297 doi: 10.1186/1475-2875-13-297 25086861 10.1186/1475-2875-13-297 PMC4127654

[11] Toni T, Welch D, Strelkowa N, Ipsen A, Strumpf MP. Approximate Bayesian computation scheme for parameter inference and model selection in dynamical systems. Journal of the Royal Society Interface. 2009; 6: 187–202. doi: 10.1098/rsif.2008.0172 10.1098/rsif.2008.0172 PMC2658655 19205079

[12] Ghasemi O, Lindsey ML, Yang T, Nguyen N, Huang Y, Jin Y. Bayesian parameter estimation for nonlinear modelling of biological pathways. BMC Systems Biology. 2011; 5:S9 Available from: http://www.biomedcentral.com/1752-0509/5/S3/S9 22784628 10.1186/1752-0509-5-S3-S9 PMC3287577

[13] Huang Y, Liu D, Wu H. Hierarchical Bayesian methods for estimation of parameters in a longitudinal HIV dynamic system. Biometrics. 2006; 62: 413–423. doi: 10.1111/j.1541-0420.2005.00447.x 16918905 10.1111/j.1541-0420.2005.00447.x PMC2435289

[14] Kwong GPS, Deardon R. Linearized forms of individual-level models for large-scale spatial infectious disease systems. Bulletin of Mathematical Biology. 2012; 74: 1912–1937. doi: 10.1007/s11538-012-9739-8 22718395 10.1007/s11538-012-9739-8

[15] Malik R, Deardon R, Kwong GPS. Parameterizing spatial models of infectious disease transmission that incorporate infection time uncertainty using sampling-based likelihood approximations. PLoS ONE. 2016; 11: e0146253 https://doi.org/10.1371/journal.pone.0146253 26731666 10.1371/journal.pone.0146253 PMC4701410

[16] Putter H, Heisterkamp SH, Lange JMA, De Wolf F. A Bayesian approach to parameter estimation in HIV dynamical models. Stat. Med. 2002; 21: 2199–2214. doi: 10.1002/sim.1211 12210633 10.1002/sim.1211

[17] White MT, Conteh L, Cibulski R, Ghani AC. Costs and cost-effectiveness of malaria control interventions a systematic review. Malaria Journal. 2011; 10: 10–337. doi: 10.1186/1475-2875-10-337 22050911 10.1186/1475-2875-10-337 PMC3229472

[18] Chiyaka C, Tchuenche JM, Garira W, Dube S. A mathematical analysis of the effects of control strategies on the transmission dynamics of malaria. Applied Mathematics and Computation. 2008; 195: 641–662. doi: 10.1016/j.amc.2007.05.016

[19] Wallace DI, Southworth BS, Shi X, Chipman JW, Githeko JW. A comparison of five malaria transmission models: benchmark tests and implications for disease control. Malaria Journal. 2014; 13: 268 doi: 10.1186/1475-2875-13-268 25011942 10.1186/1475-2875-13-268 PMC4105118

[20] Koella JC. On the use of mathematical models of malaria transmission. Acta Tropica. 1991; 49: 1–25. doi: 10.1016/0001-706X(91)90026-G 1678572 10.1016/0001-706x(91)90026-g

[21] Killeen GF, Smith TA, Ferguson HM, Mshinda H, Abdulla S, et al Preventing childhood malaria in Africa by protecting adults from mosquitoes with insecticide-treated nets. PLoS Med. 2007 4: e229 doi: 10.1371/journal.pmed.0040229 17608562 10.1371/journal.pmed.0040229 PMC1904465

[22] Ngonghala CN, Del Valle SY, Zhao R, Awel JM. Quantifying the impact of decay in bed-net efficacy on malaria transmission. Journal of Theoretical Biology. 2014; 363: 247–261. doi: 10.1016/j.jtbi.2014.08.018 25158163 10.1016/j.jtbi.2014.08.018 PMC4374367

[23] Okell LC, Drakeley CJ, Bousema T. Modelling the impact of artemisinin combination therapy and long-acting treatments on malaria transmission intensity. PLoS Medicine. 2008; 5: 1617–1628. doi: 10.1371/journal.pmed.0050226 10.1371/journal.pmed.0050226 PMC2586356 19067479

[24] Smith DL, McKenzie FE, Snow RW, Hay SI. Revisiting the basic reproductive number for malaria and its implications for malaria control. PLoS Biol. 2007; 5: 42–25. doi: 10.1371/journal.pbio.0050042 10.1371/journal.pbio.0050042 PMC1802755 17311470

[25] Ngonghala CN, Awel JM, Zhao R, Prosper O. Interplay between insecticide-treated bed-nets and mosquito demography:implications for malaria control. Journal of Theoretical Biology. 2016; 397: 179–192. doi: 10.1016/j.jtbi.2016.03.003 26976050 10.1016/j.jtbi.2016.03.003

[26] Chitnis N, Schapira A, Smith T, Steketee R. Comparing the effectiveness of malaria vector-control interventions through a mathematical model. The American Society of Tropical Medicine and Hygiene. 2010; 83: 230–240. doi: 10.4269/ajtmh.2010.09-0179 10.4269/ajtmh.2010.09-0179 PMC2911164 20682861

[27] Okumu FO, Kiware SS, Moore SJ, Killeen GF. Mathematical evaluation of community level impact of combining bed nets and indoor residual spraying upon malaria transmission in areas where the main vectors are Anopheles arabiensis mosquitoes. Parasites Vectors. 2013; 3: Available from: http://www.parasitesandvectors.com/content/6/1/17 10.1186/1756-3305-6-17 PMC3564902 23324456

[28] Briët OJT, Penny MA. Repeated mass distributions and continuous distribution of long-lasting insecticidal nets: modelling sustainability of health benefits from mosquito nets, depending on case management. Malaria Journal. 2013; 12: 12–401. 24200296 10.1186/1475-2875-12-401 PMC4228503

[29] Griffin JT, Hollingsworth TD, Okell LC, Churcher TS, White M, Hinsley W, et al Reducing Plasmodium falciparum Malaria Transmission in Africa: A Model-Based Evaluation of Intervention Strategies. PLoS Medicine. 2010; 8. 10.1371/journal.pmed.1000324 PMC2919425 20711482

[30] Filipe JAN, Riley EM, Drakeley CJ, Sutherland CJ, Ghani AC. Determination of the processes driving the acquisition of immunity to malaria using a mathematical transmission model. PLOS Computational Biology. 2007); 3: 2569–2579. doi: 10.1371/journal.pcbi.0030255 10.1371/journal.pcbi.0030255 PMC2230683 18166074

[31] SSCCSE: South Sudan counts: Tables from the 5th Sudan population and housing census, 2008. Juba: Government of Southern Sudan: Southern Sudan Centre for Census: Statistics and Evaluation; 2010.

[32] South Sudan malaria programme review report 2013.

[33] South Sudan Malaria Strategic Plan 2014/15-2020/21.

[34] White MT, Griffin JT, Churcher TS, Ferguson NM, Basanez MG, Ghani AC. Modelling the impact of vector control interventions on Anopheles gambiae population dynamics. Parasit Vectors. 2011; 4: 153–337. doi: 10.1186/1756-3305-4-153 21798055 10.1186/1756-3305-4-153 PMC3158753

[35] Smith DL, Hay SI, Noor AM, Snow RW. Predicting changing malaria risk after expanded insecticide-treated net coverage in Africa. Trends Parasitol. 2009; 25: 511–516. doi: 10.1016/j.pt.2009.08.002 19744887 10.1016/j.pt.2009.08.002 PMC2768685

[36] Dræbel T, Kueil BG, Meyrowitsch DW. Prevalence of malaria and use of malaria risk reduction measures among resettled pregnant women in South Sudan. International Health. 2013; 5: 211–216. doi: 10.1093/inthealth/iht008 24030272 10.1093/inthealth/iht008

[37] Van den Driessche P, Watmough J. Reproduction numbers and sub threshold endemic equilibria for compartmental models of disease transmission. Mathematical Biosciences. 2002; 180: 29–48. doi: 10.1016/S0025-5564(02)00108-6 12387915 10.1016/s0025-5564(02)00108-6

[38] Hamra G, MacLehose R and Richardson D. Markov Chain Monte Carlo: an introduction for epidemiologists. International Journal of Epidemiology. 2013; 42: 627–634. doi: 10.1093/ije/dyt043 23569196 10.1093/ije/dyt043 PMC3619958

[39] Soetaert K. and Petzoldt T. Inverse Modelling, Sensitivity and Monte Carlo Analysis in R Using Package FME. Journal of Statistical Software. 2010; 33: 3.

[40] Camacho A, Funk S. FitR: Tool box for fitting dynamic infectious disease models to time series. (2017) Version 0.1 Available from: https://github.com/sbfnk/fitR.

[41] Sovi A, Azondékon R., Aíkpon RY, Govoétchan R, Tokponnon F, Agossa F, et al Impact of operational effectiveness of long-lasting insecticidalnets (LLINs) on malaria transmission in pyrethroid-resistant areas. Parasit Vectors. 2013; 6: 319 doi: 10.1186/1756-3305-6-319 24499508 10.1186/1756-3305-6-319 PMC4029312

[42] Griffin JT, Ferguson NM and Ghani AC. Estimates of the changing age-burden of Plasmodium falciparum malaria disease in sub-Saharan Africa. Nat. Commun. 2014; 5:31–36. doi: 10.1038/ncomms4136 10.1038/ncomms4136 PMC3923296 24518518

[43] Le Menach A, Takala S, McKenzie FE, Perisse A, Harris A, Flahault A, et al An elaborated feeding cycle model for reductions in vectorial capacity of night-biting mosquitoes by insecticide-treated nets. Malaria Journal. 2007; 10: 6–10. 10.1186/1475-2875-6-10 PMC1794417 17254339

