# Supplementary material for: Modelling the effect of bednet coverage on malaria transmission in South Sudan
Source: PLoS One. 2018 Jun 7;13(6):e0198280. doi: 10.1371/journal.pone.0198280 (PMC5991726; doi:10.1371/journal.pone.0198280)
Supplement: S1 Appendix — (PDF) [file pone.0198280.s001.pdf]

Proof of the proportion 4.1
Set $x = (I, A, E, S)^{\mathrm{T}}$, with W^{T} being the transpose of the vector W. Then the system can be written as

$$\dot{x} = \mathcal{F}(x) - \mathcal{V}(x)$$

where

$$\mathcal{F}(x) = \begin{pmatrix} \frac{\mu_v \epsilon b I_v S}{\psi} \\ 0 \\ 0 \\ \frac{\sigma_a \epsilon A X + \sigma_i \epsilon I X}{\Gamma} \\ 0 \end{pmatrix},$$

$$\mathcal{V}(x) = \begin{pmatrix} (\eta + \rho + \mu)E \\ (\mu + \alpha_1)A - \rho E \\ (\mu + \delta + \alpha_2)I - \eta E \\ (\beta + \mu_v)E_v \\ -\beta E_v + \mu_v I_v \end{pmatrix}.$$

According to the theory of [1], the basic reproduction number $\mathcal{R}_0$ of our system is the spectral radius of FV^{-1}, where F and V are the matrices

$$F = \begin{pmatrix} 0 & 0 & 0 & 0 & \frac{\mu_v \epsilon b \Gamma}{\mu \psi} \\ 0 & 0 & 0 & 0 & 0 \\ 0 & 0 & 0 & 0 & 0 \\ 0 & 0 & 0 & 0 & 0 \\ 0 & \frac{\sigma_i \epsilon \mu \psi}{\mu_v \Gamma} & \frac{\sigma_a \epsilon \mu \psi}{\mu_v \Gamma} & 0 & 0 \\ 0 & 0 & 0 & 0 & 0 \end{pmatrix}$$

and

$$V = \begin{pmatrix} (\eta + \rho + \mu) & 0 & 0 & 0 & 0 \\ -\rho & (\alpha_1 + \mu) & 0 & 0 & 0 \\ -\rho & 0 & (\alpha_2 + \delta + \mu) & 0 & 0 \\ 0 & 0 & 0 & (\beta + \mu_v) & 0 \\ 0 & 0 & 0 & -\beta & \mu_v \end{pmatrix}.$$

The matrix F is a non-negative matrix of rank one and can be written as the product of the vectors, where V is a non-singular M-matrix. The inverse of V is

$$V^{-1} = \begin{pmatrix} \frac{1}{\eta+\rho+\mu} & 0 & 0 & 0 & 0 \\ \frac{-\eta+}{(\eta+\rho+\mu)(\alpha_2+\delta+\mu)} & \frac{1}{\alpha_2+\delta+\mu} & 0 & 0 & 0 \\ \frac{\rho}{(\eta+\rho+\mu)(\alpha_1+\mu)} & 0 & \frac{1}{\alpha_1+\mu} & 0 & 0 \\ 0 & 0 & 0 & \frac{1}{\gamma+\mu_v} & 0 \\ 0 & 0 & 0 & \frac{-\beta}{(\beta+\mu_v)\mu_v} & \frac{1}{\mu_v} \end{pmatrix}.$$

Multiplying F and V^{-1} gives the next generation matrix

$$G := FV^{-1} = \begin{pmatrix} 0 & 0 & 0 & k_1 & k_2 \\ 0 & 0 & 0 & 0 & 0 \\ 0 & 0 & 0 & 0 & 0 \\ 0 & 0 & 0 & 0 & 0 \\ k_3 & k_4 & k_5 & 0 & 0 \\ 0 & 0 & 0 & 0 & 0 \end{pmatrix}, \tag{5.3}$$

where

$$k_1 = \frac{\beta\epsilon b\Gamma}{(\beta+\mu_v)\mu\psi}$$

$$k_2 = \frac{\epsilon\Gamma\sigma_i}{\mu\psi}$$

$$k_3 = \frac{\eta\sigma_i\mu\epsilon\psi}{(\eta+\rho+\mu)(\alpha_2+\delta+\mu)\mu_v\Gamma} + \frac{\rho\sigma_a\epsilon\mu\psi}{(\eta+\rho+\mu)(\alpha_1+\mu)\mu_v\Gamma}$$

$$k_4 = \frac{\sigma_i\mu\epsilon\psi}{(\alpha_2+\delta+\mu)\mu_v\Gamma}$$

$$k_5 = \frac{\sigma_a\epsilon\mu\psi}{(\alpha_1+\mu)\mu_v\Gamma}$$

Hence we compute the eigenvalues to obtain the spectral radius of the matrix FV^{-1}. The spectral radius is the reproductive number $\mathcal{R}_0$. There are five eigenvalues obtained from FV^{-1} and maximum eigenvalue is $\lambda = \sqrt{k_1 k_3}$. Therefore the basic reproduction number for the system is as claimed. □

References

[1] Van den Driessche P, Watmough J. Reproduction numbers and sub threshold endemic equilibria for compartmental models of disease transmission. Mathematical Biosciences. 2002; 180: 29-48.
